# Gene Regulation Systems for Gene Therapy Applications in the Central Nervous System

**DOI:** 10.1155/2012/595410

**Published:** 2012-01-05

**Authors:** Jerusha Naidoo, Deborah Young

**Affiliations:** Department of Pharmacology and Clinical Pharmacology and Centre for Brain Research, University of Auckland, Auckland 1023, New Zealand

## Abstract

Substantial progress has been made in the development of novel gene therapy strategies for central nervous system (CNS) disorders in recent years. However, unregulated transgene expression is a significant issue limiting human applications due to the potential side effects from excessive levels of transgenic protein that indiscriminately affect both diseased and nondiseased cells. Gene regulation systems are a tool by which tight tissue-specific and temporal regulation of transgene expression may be achieved. This review covers the features of ideal regulatory systems and summarises the mechanics of current exogenous and endogenous gene regulation systems and their utility in the CNS.

## 1. Introduction

Recent years have seen a plethora of potential gene therapy strategies for central nervous system (CNS) disorders. One of the major challenges gene therapy applications face clinically is the ability to control the level of expression or silencing of therapeutic genes in order to provide a balance between therapeutic efficacy and nonspecific toxicity due to overexpression of therapeutic protein or RNA interference-based sequences. Thus, the ability to regulate gene expression is essential as it reduces the likelihood of potentially initiating adverse events in patients. Although genes may be regulated at either the translational or posttranscriptional level, greatest success in gene regulation has been at the transcriptional level and as such gene regulation systems at a transcriptional level is the focus of this review. There are two classes of gene regulation systems—exogenously controlled gene regulation systems, which rely on an external factor (usually the administration of a drug) to turn transgene expression on or off, and endogenously controlled gene expression systems that rely on physiological stimuli to control transgene expression. This review covers the characteristics of an ideal regulatory system and summarises the mechanics of current gene regulation systems and their application to CNS disorders.

## 2. What Are the Characteristics of a Good Gene Regulation System?

In order to be clinically effective, it has been proposed that regulatory systems should possess the following characteristics [1]:

- *Exhibit low basal expression*. In the “off” state, transgene expression should be subphysiological. This would be crucial in the event of an adverse reaction to the treatment.
- *Be positively induced*. Induction of gene expression should be positive, through the presence, rather than the absence of an inducing stimulus so that patients can discontinue drug therapy when treatment is no longer required. In the case of endogenously controlled systems, this would enable transgene expression to be repressed when the treatment is no longer required.
- *Transgenic protein levels should lie between therapeutic indices*. Transgene expression should be inducible to physiological levels to enable a therapeutic outcome.
- *Be regulatable over a wide dose range*. Responsiveness to a wide dose range of the inducer will enable greater precision in titrating transgene expression levels to optimal therapeutic levels.
- *Exert no pleiotropic effects*. Ideally the transgene would produce the desired effect on its cellular targets and not have an effect on any other phenotypic traits; however, this may not be controllable.
- *Be of human origin*. The risk of immunogenicity is decreased when components of regulatory systems are of human origin.
- *Not affect endogenous gene expression*. Components of the regulatory system such as transcription factors or small molecule inducers should not have pleiotropic effects in mammalian cells and affect expression of endogenous genes.
- *Be region or cell specific*. It would be efficacious to limit transgene expression to specific regions or cell types that require it.
- *Transgene expression should be induced and repressed as quickly as possible.* This would enable maximal physiological benefit to be obtained and would (i) reduce the likelihood of toxic overexpression of transgene in cells that no longer require it and (ii) enable discontinuation of adverse effects to the treatment in a timely manner.

## 3. Exogenously Regulated Gene Expression Systems

Exogenously regulated systems are the most well-characterised class of regulation systems, and substantial progress has been made on their design and optimization in recent years. This section covers the mechanics and utility of popular systems for CNS gene therapy (see Table 1 for a summary of *in vivo* applications).

### 3.1. Tetracycline Regulated Systems

The tetracycline regulated gene expression system developed by Gossen and Bujard [2] is the most widely used gene regulation system. There are two variants: the Tet-Off system was the first to be developed [2], followed by the Tet-On system [3], that has now become more popular. The Tet-Off system is negatively controlled and relies on tetracycline to deactivate expression, whereas the Tet-ON system relies on tetracycline to activate gene expression.

**Figure 1:**
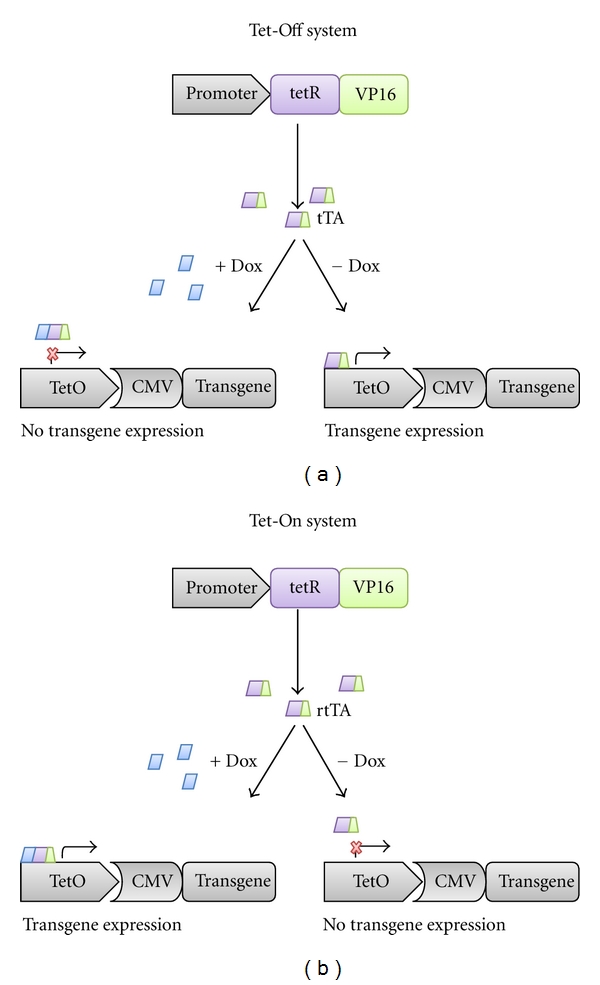
Mechanics of tetracycline regulated systems. A constitutively active promoter drives expression of tetracycline transactivators (tTAs) or reverse tetracycline transactivators (rtTAs). (a) *Tet-Off* system. tTAs are able to bind to the tet-operator sequence (tetO) in the absence, but not in the presence of doxycycline (dox) to drive transgene expression. (b) *Tet-On* system. rtTAs are able to bind to the TRE in the presence, but not in the absence of dox to drive transgene expression. tetR: tetracycline repressor; VP16: viral protein 16; CMV: cytomegalovirus.

Transactivators (tTAs) are constitutively produced by a cell or tissue-specific promoter through fusion of a viral protein (VP16) domain to the tet-repressor protein. In the Tet-Off system, when doxycycline, a tetracycline derivate is present, the transactivators are unable to bind to their target, a tet-operator sequence upstream of a cytomegalovirus (CMV) promoter (termed the tetracycline response element [TRE]) that drives transgene expression. Conversely, in the absence of doxycycline, the tTAs bind to the TRE, driving transgene expression (Figure 1(a)). The Tet-On system was developed by inducing random mutations in the tTA of the Tet-Off system [3]. One of the mutations led to a four amino-acid change that resulted in a protein with opposite function. These mutant proteins, termed reverse transactivators (rtTAs), drive transgene expression by activating the TRE only in the presence of doxycycline. Absence of doxycycline results in the inhibition of transgene expression (Figure 1(b)).

Tetracycline-based regulation systems are a good candidate for gene therapy applications due to their extensive characterization and the fact that tetracycline and its derivatives have been used clinically for decades. The majority of CNS applications involving this system have used viral vectors for construct delivery, of which adenoassociated viral (AAV) vectors have been the most popular.

**Figure 2:**
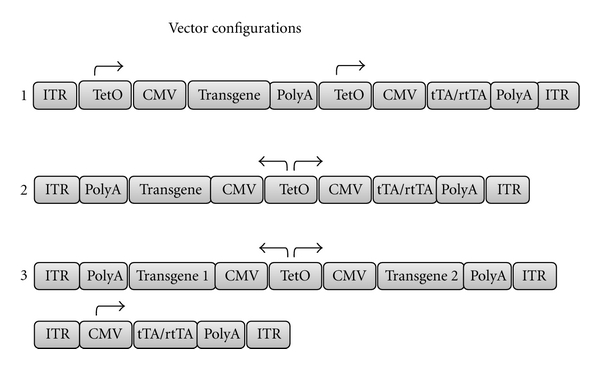
Different configurations the tetracycline system has been incorporated into viral vectors for use in the brain. (1) Two right-facing cistrons direct transactivator and transgene expression in a single vector [4–7]. (2) One bidirectional promoter driving expression of the transgene and transactivators in a single construct [5, 6, 8–10]. (3) Two vectors are used: one bidirectional construct directing expression of two transgenes and one driving expression of transactivators [11, 12]. ITR: inverted terminal repeats; PolyA: polyadenylation signal; TetO: tetracycline operator sequence; CMV: cytomegalovirus; tTA: tetracycline transactivator; rtTA: reverse tetracycline transactivator.

Haberman and colleagues were the first to demonstrate that AAV vectors coupled with the Tet-Off system can be used to produce regulated, long-term gene expression in the rat brain [4]. A dual cassette was constructed with the gene-encoding enhanced green fluorescent protein (GFP) under the control of the TRE and a gene-encoding modified tTAs on the same construct. The tTAs and gene of interest were placed under the same transcriptional control to limit leakiness and decrease the likelihood of cytotoxic effects of tTAs. This method was dependent on the activity of the CMV promoter to produce a small amount of tTA protein to initiate a feedback loop to enhance tTA and therefore transgene expression. AAV-expressing constructs were injected into the inferior colliculus, and transgene regulation was achieved through the presence or absence of doxycycline in the drinking water. Since this pioneering study, a number of other groups have incorporated the tetracycline system into viral vectors for both *in vitro* and *in vivo* applications. There are five main arrangements that have been used, using either one or two vectors (Figure 2).

AAV vectors have been the most widely used, followed by adenoviral vectors [6, 12] and lentiviral (LV) vectors [13]. Single vector designs limit the size of the transgene that can be incorporated; AAV vectors have a maximum capacity of approximately 5.0 kb, leaving approximately 2.0–2.5 kb for transgenes. However, the tetracycline system is small in comparison to other systems, and some groups have managed to incorporate multiple transgenes into a single-vector construct [5, 9, 11]. A two-vector design, where the tTA/rtTAs are encoded on a separate vector, enables transgenes of up to 4.0 kb to be incorporated into a single AAV vector. This design requires both vectors to transduce each cell in order to be functional, so the ratio of vectors must be tightly controlled. Single-vector designs that utilize constitutive promoters such as CMV suffer from interference between the tet-operator sequence and the CMV promoter, leading to high basal transgene expression. This issue has been addressed by placing the tTA/rtTAs under the control of a weak minimal promoter such as the thymidine kinase promoter [8] or the tetO sequence itself [14, 15].

Although it is difficult to make direct comparisons due to the number of different construct compositions used, in terms of kinetics, high inducibility and repression has been achieved both *in vitro* and *in vivo*; however, there is still some residual transgene expression present in the “off” state. The induction of transgene expression should be as quick as possible in order to facilitate maximal physiological benefit. Maximal transgene expression has been reported within 48 h *in vitro* [9, 11]. Additionally, transgene expression can be induced *in vivo* as early as 6 days [6], with levels as high as 80 and 204-fold [5, 8]. Whether the time to induction reported in these studies is acceptable will depend on the disease being treated. Neurodegenerative disorders progress slowly so the forementioned induction times are probably acceptable. However, acute conditions such as stroke necessitate prompt action so these time periods would not be ideal.

Repression can be achieved quite rapidly—as early as 2 days *in vitro* [11] and 3–10 days *in vivo* [5, 9]. Whether this time period is satisfactory will depend on the toxicity profile of the transgene being expressed and/or the severity and nature any adverse reaction to the treatment. Tight regional distribution of transgene has been achieved in the rat brain using the AAV/Tet-On system, compared to the constitutively active AAV/CMV system [16]. To ensure cell-specific transgene expression, the CMV promoter has been replaced with the neuron-specific enolase (NSE) promoter [13]. Additionally, physiologically relevant levels of transgenic protein are being produced in the brain—of particular note is a study conducted by Manfredsson and colleagues who used an AAV/Tet-Off system to express glial cell line-derived neurotrophic factor at levels exceeding those required for efficacy in a 6-hydroxydopamine model of Parkinson's disease [7]. More recently, doxycycline-controlled expression of cell-specific microRNA sequences that mediate RNA interference-based gene silencing has also been demonstrated broadening the utility of this system in regulating another potential class of therapeutic molecule [17].

In order to attain adequate dose-responsive control and limit the likelihood of toxic overexpression of the transgene, it is desirable to use the lowest possible concentration of doxycycline. Although doxycycline has a high serum uptake and clearance, it has a considerably slow tissue clearance. This means that transgene activation is an inherently faster process than repression. Consequently, the Tet-On system would be more suitable for conditions with acute onset such as stroke, where transgene activation is required rapidly; however, this would be accompanied with the risks mentioned above. For all other applications, the Tet-Off system would be a safer option but would require life-long doxycycline administration following cessation of the treatment. Moreover, it would be important to establish that long-term expression of tTA protein also causes no toxicity. Of note, no humoral immune response against the tTA protein was found following intrastriatal infusion of a tet-off AAV vector expressing hAADC or hGDNF suggesting that these might be safer vectors for Parkinson's disease gene therapy [18].

 There are two main issues that are thought to contribute to the leakiness of the tetracycline system in the off state. The first is that the transactivators retain some ability to bind to the TRE in the uninduced state, and the second is the close proximity of the TRE to the inverted terminal repeats, which possess enhancer activity [19]. Considerable research has been undertaken to reduce the likelihood of transactivators binding to tet-operator sequence under basal conditions. Urlinger and colleagues have developed two systems: one involved the identification of novel rtTAs through PCR mutagenesis of the tetracycline repressor protein. To do this, PCR of original rtTAs was conducted under conditions designed to induce misincorporation of nucleotides. Compared to the original rtTA, modified rtTA was associated with considerably lower background expression, while its activation potential was nearly the same as the unmodified rtTA [20]. The second strategy involved producing a transcriptional silencer which binds to promoters responsive to rtTA and blocks their activation in the absence of doxycycline. In the presence of doxycycline, transcriptional silencers are unable to bind to the promoter, relieving transcriptional repression and promoting binding of rtTA which enables transgene expression. Combining the forementioned systems in a bicistronic construct reduced basal expression 6-fold compared to expression of the novel transactivators alone; however, activation was approximately 3-fold less potent with these bicistronic constructs compared to their monocistronic counterparts [21].

To circumvent the issue of inverted terminal repeat proximity, Fitzsimons and colleagues have inserted insulators flanking the regulation system. While the leakiness was only partially ameliorated *in vitro*, repression was increased from 40 to 204-fold *in vivo* [8]. A bidirectional composition, where the TRE is located in the middle of the construct, maximizes the distance from the inverted terminal repeats and appears to produce the least amount of basal expression; however, there is still a considerable amount of transgene expression present. Other hypotheses to explain basal expression include the integration of transgenes at unsuitable chromosomal sites or close proximity of the transgene to the element driving transactivator expression [22].

These findings suggest that vector-mediated delivery of tetracycline-based regulatory systems shows considerable promise in the treatment of CNS disorders; however, additional studies are required to further optimize this system to reduce basal expression whilst maintaining high induction levels.

### 3.2. Rapamycin-Regulated Systems

**Figure 3:**
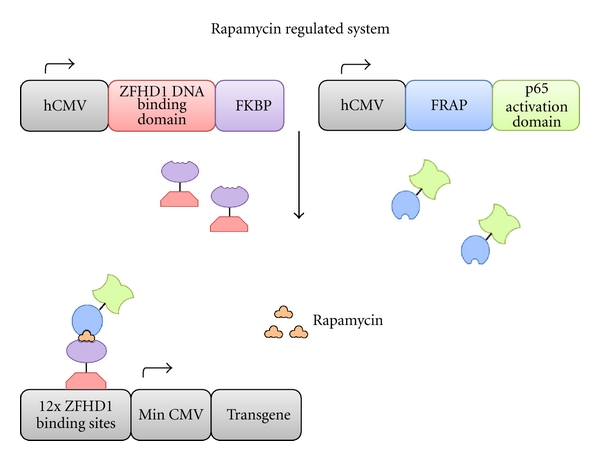
Schematic of the rapamycin regulation system. The constitutively active human cytomegalovirus (hCMV) promoter drives expression of two fusion transcription factors. (Top left) A transcription factor consisting of three copies of the FKBP protein fused to a ZFHD1 DNA binding domain. (Top right) A transcription factor consisting of a FRAP protein with a p65 activation domain. Rapamycin enables dimerization of the transcription factors, with enable binding to 12xZFHD1 binding sites and activation, respectively, driving expression of a transgene upstream of a minimal CMV promoter. ZFHD1: zinc finger homeodomain-1; FKBP: FK-binding protein; FRAP: FKBP-rapamycin associated protein [23].

Designed by Rivera and colleagues [23], the rapamycin-regulated gene regulation system relies on the interaction between two transcription factors, one incorporating a DNA-binding domain and the other a DNA activation domain. Each of the transcription factors also contains a heterologous ligand-binding domain that enables their interaction in the presence of the dimerizing drug rapamycin to drive transgene expression. DNA binding is facilitated through the human CMV promoter driven production of a zinc finger homeodomain-1 (ZFHD1) DNA-binding domain fused to three copies of the FK-binding protein (FKBP). Transgene expression is achieved in the presence of rapamycin, which induces dimerization of this DNA-binding protein with a fusion protein consisting of the FKBP-rapamycin-associated protein 1 (FRAP) fused to the NF*κ*B p65 activation domain (Figure 3).

Due to the size of this system, two viral vectors are required for delivery of all the components. A 1 : 1 ratio of transcription factor vector to transgene vector is sufficient for high induction and low basal transgene expression [24]. This system has many of the properties required for use clinically. It is characterized by a high induction ratio, low basal expression, and is composed entirely of human proteins. Additionally, rapamycin can be administered orally and has a pharmacokinetic profile that has been widely studied. The primary issue with this system was that rapamycin functions as an immunosuppressant through blocking FRAP activity [25] and inhibiting progression through the cell cycle at concentrations required for gene regulation. Rapamycin analogs (“rapalogs”) have since been engineered by adding substituents which prevent binding to FRAP [26].

Only a few studies have tested this system in the CNS using viral vector delivery thus far, leaving this field largely uncharacterized. Rapalogs have been used to increase expression of human aromatic L-amino acid decarboxylase (hAADC), the enzyme that converts levodopa to dopamine in the striatum of a rodent model of Parkinson's disease (PD) [24]. AAV vectors were coinfused, one expressing the transcription factors and one encoding the hAADC transgene downstream from a rapamycin-inducible promoter. Rapamycin-induced robust rotational behaviour in the presence of low doses of levodopa and although low levels of hAADC were observed in the absence of rapamycin, these levels were not significant enough to induce a behavioural response. This study demonstrated for the first time that rapamycin is able to efficiently cross the blood-brain barrier and induce expression of recombinant protein at levels that have a significant phenotypic effect, and in a sufficiently regulated manner. More recently LV-mediated transfer of a rapalog-controlled system has been used to tightly regulate expression of glial cell line-derived neurotrophic factor and GFP in the striatum of mice [27]. It is yet to be established whether LV vectors can be used to produce recombinant proteins at physiologically relevant levels using the rapamycin system. Further studies are required to gain a thorough dose-responsive profile of rapalogs in the CNS and to explore the possibility of using cell-specific promoters such as the tyrosine hydroxylase or NSE promoters for more targeted transgene expression. Additionally, given that the proteins in this system are of human origin, it would be prudent to investigate the possible effects of these chimeric proteins on endogenous gene expression in the brain.

### 3.3. RU486-Regulated System

**Figure 4:**
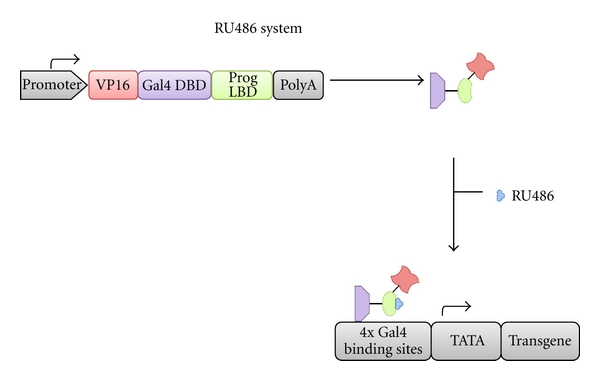
Schematic of the RU486 regulation system. A constitutively active promoter drives expression of a fusion protein consisting of a VP16 activation domain, a Gal4 DNA-binding domain (DBD) and a progesterone ligand-binding domain (Prog LBD). Binding of RU486 to the Prog LBD enables dimerization and binding to 4x Gal 4-binding sites upstream of a TATA box driving transgene expression [28]. Poly A, poly adenylation signal.

The RU486 system is conceptually similar to the rapamycin system; it relies on the drug RU486 to activate a chimeric transcription factor that drives transgene expression. A chimeric regulator protein is constitutively produced, consisting of the DNA-binding domain of the yeast transcription factor Gal4 (Gal4 DBD), a truncated progesterone ligand-binding domain (PRLBD), and a VP16 activation domain. In the presence of RU486, the regulator protein is activated, enabling binding to the target construct consisting of 17x4Gal4 DNA-binding sites and a minimal promoter (such as TATA) upstream of the transgene (Figure 4). Tissue-specific promoters can be used to produce the transcription factor [28].

The RU486 system has been incorporated into adenoviral, LV, and herpes simplex viral (HSV) vectors but has only been tested *in vivo* in one study thus far. It is similar in size to the tetracycline regulated system and may be incorporated into one or two vectors (in a 1 : 1 ratio [29]) depending on the size of the transgene to be expressed. The *in vivo* study involved using a single HSV viral vector to deliver a bicistronic construct expressing both the LacZ reporter gene under the control of a minimal Gal4 promoter and the regulator protein under the control of a minimal hCMV promoter to the rat hippocampus. Upon induction via intraperitoneal injection of RU486, *β*-galactosidase levels increased 150-fold compared to basal levels, which were extremely low [30]. Interestingly, results from *in vitro* studies involving viral vectors have not been as consistent. Molin and colleagues compared the efficacy of the RU486 system with the Tet-On system when packaged into Ad vectors. A two-vector system was used, with one vector encoding the reporter gene chloramphenicol acetyltransferase (CAT) and the other encoding the respective transcription factors. While the RU486 system-induced CAT expression to high levels in three other cell lines, it failed to induce sufficient CAT levels in the NIH 3T3 cell line. Given that the Tet-On system worked well in this cell line, it is likely that this failure was due to the inability of RU486 to activate the progesterone receptor in this cell type, rather than inefficient vector uptake [31]. Additionally, a more recent *in vitro* study involving LV delivery of the RU486 system showed robust induction of red fluorescent protein and GFP, but not of antitrypsin protein [29]. The authors suggest that the RU486 system may function differently when expressing secretable versus intracellular proteins. Relatively low concentrations of RU486 have been used to achieve robust transgene expression *in vitro *(10^−7^–10^−8^ M) [29, 30]. However, the dose given to induce transgene expression *in vivo* (25 mg/kg) exceeds the dose used clinically as an antiprogesterone therapy (10 mg/kg). Given this data, a considerable amount of additional investigation needs to be undertaken before the RU486 system can be used clinically for the treatment of CNS disorders. Specifically, information is required on the dose-responsiveness of RU486 *in vivo* and the time required for transgene repression following the removal of RU486. Lastly, it will be imperative to determine whether the RU486 system can produce physiologically relevant levels of transgenic protein in the brain, as no phenotypic studies have been undertaken.

## 4. Endogenously Controlled Gene Expression Systems

Physiologically responsive gene expression systems incorporate negative feedback loops, where the recombinant protein shuts off its own production in response to the presence or absence of a physiological signal. Since the focus of this review is gene regulation in the CNS, only those physiologically regulated systems with potential for application to the CNS will be covered (see Table 1 for a summary of *in vivo* applications); however, it should be noted that there has been robust success in the field of glucose-responsive control of insulin production [32, 33].

### 4.1. Hypoxia-Regulated System

Oxygen deprivation or hypoxia is a common feature of neuropathophysiological conditions such as stroke and cerebral ischemia. The physiological response to hypoxia includes induction of several genes mediated by the transcription factor HIF1 (hypoxia-inducible factor 1). HIF1 is composed of two subunits: HIF1*α* and HIF1*β*. Under hypoxic conditions, HIF-1*α* is produced and dimerizes with HIF-1*β*, which is constitutively produced under normoxic conditions, forming HIF1, which binds to hypoxia response elements (HREs) inducing transcription of a downstream gene of interest. HIF1*α* levels increase in neurons, astrocytes, ependymal, and possibly endothelial cells in response to hypoxia in the brain [34]. In terms of a therapeutic strategy, hypoxia-regulated gene expression is achieved by the insertion of multiple copies of the HRE upstream of a promoter that drives transgene expression. 

This system has been incorporated into viral vectors for use in the brain and shows considerable promise as a strategy for the treatment of cerebral ischemia. It is associated with low transgene expression under normoxic conditions that is inducible in a hypoxia-dependent manner in both *in vitro* and *in vivo* models. Studies in mouse models of cerebral ischemia have demonstrated increases in transgene levels of approximately 17- [35] and 28-fold [36] following adenoviral gene delivery. Whether or not these levels are sufficient to be therapeutic depends on the candidate gene and the duration of transgene expression. Expression levels of brain-derived neurotrophic factor and vascular endothelial growth factor were shown to be sufficient to elicit a phenotypic response in mouse models of cerebral ischemia, by promoting neuroprotection and angiogenesis, respectively [36–38]. Achieving a rapid response will be pivotal in a clinical setting; however, information is lacking on the temporal nature of transgene expression using this system. Transgene expression has been reported as early as 12 h following hypoxia *in vivo* [36]; however, it is possible that this response might be initiated at an earlier time point. Interestingly, *in vitro* transgene expression has been reported at 3 hours in a serum deprivation-induced model of hypoxia in PC12 cells [39]. Accordingly, it is important that transgene expression is repressed in a timely manner after hypoxia to reduce the likelihood of toxic overexpression of transgenic protein. Shen and colleagues have demonstrated that transgenic protein persists in the brain for 2 weeks after hypoxia [37]; however, the upper limit may be longer than this. Given that activation and repression of transgene expression is dependent on changes in oxygen (and thus HIF1*α*) levels, as the duration and severity of hypoxia increases, there should be a concomitant increase in transgene expression. Indeed, a duration-dependent increase in transgene levels has been demonstrated in PC12 cells following hypoxia treatment for 1, 3, 6, 9, 12, and 24 h [39, 40]. Additionally, Huang and colleagues have demonstrated a severity-dependent increase in transgene expression following treatment with 0.1, 1, and 5% oxygen levels in PC12 cells [40].

Shen and colleagues have found that AAV vector delivery of this system results in transgene expression in both neurons and astrocytes [37]. In order to facilitate selective targeting of neurons, neuron-specific promoters could be used; however, studies thus far have only utilized minimal constitutively active promoters. Interestingly, Hou and colleagues have described a different approach to neuronal targeting; they have incorporated a neuron-restrictive silencer element into the construct [35, 40]. The neuron-restrictive silencer element is controlled by a factor that is only present in nonneuronal cells [41]. Neuronal-specific targeting has been achieved both *in vitro* and *in vivo* using this construct; however, this has been at the expense of the induction ratio [35].

While the majority of studies have used viral delivery methods, Ha and colleagues have employed both naked plasmid injection and *ex-vivo* stem cell delivery to rat models of spinal cord injury. Hypoxia-induced transgene expression was demonstrated in both neurons and astrocytes [42–44]. Interestingly, these studies have injected the test constructs immediately following the hypoxic insult while studies using viral vectors have injected the constructs between 5 and 7 days prior to hypoxia to enable adequate transduction. Given that minimizing the time to transgene expression is key to the development of successful therapies for cerebral ischemia using this system, there remains a need for these studies to be conducted in a more clinically relevant manner. Additionally, since HIF-1*α* is an important regulatory molecule involved in number of downstream gene networks under normoxic conditions [45], gene expression profiling needs to be conducted before HRE-regulated treatments can be widely used.

## 5. Exogenous versus Endogenous Gene Therapy

Exogenous and endogenously regulated gene therapies are each associated with a set of advantages and disadvantages when considering their use in patients. Firstly, exogenously regulated systems usually require long-term administration of a drug. This necessitates extensive characterization of the safety and tolerability of the drug, which, with the exception of tetracycline, has not occurred thus far. Additionally, it is not possible to tailor gene expression precisely to pathological signals using exogenous systems unless they are associated with a noticeable phenotypic change in a timely manner. In most cases, there is a latent period between the presence of a pathological signal and the appearance of clinical symptoms. Therefore, endogenous systems have the advantage of responding in a timelier manner and with an intensity that is tailored to the individual patient.

Additionally, gene expression systems are typically administered to whole organs or entire regions of organs, and while it may be advantageous to express transgene in this manner, this is quite often not the case. By linking transgene expression to a pathological signal, recombinant protein will only be produced in cells that require it, and toxic overexpression in healthy cells can be avoided. Conversely, a major limitation of endogenous systems is that once administered it will be virtually impossible to stop transgene expression in the event of an adverse reaction, unless the population of cells of interest is removed, or there is a built-in safety mechanism. Additionally, the pathological signals used need to be as far upstream as possible and be specific to the disease phenotype to enable transgene to be expressed in a timely and precise manner.

## 6. Conclusions

Regulation of transgene expression remains a key issue limiting widespread clinical application of gene therapies for CNS disorders. Of the exogenous regulation systems covered, tetracycline-regulated systems have been refined sufficiently such that they are on the cusp of being applied clinically. While low basal transgene expression is of concern, in a clinical setting these levels may not be sufficient to elicit a phenotypic response. Given the advances in gene delivery technology and the advantages of coupling transgene expression to a physiological signal, the focus will now be the development of novel endogenously regulated systems for use in the CNS.

## Figures and Tables

**Table 1:** Summary of use of gene regulation systems for *in vivo* CNS applications.

Regulation system (VC*)	Stimuli/dose	Delivery vehicle	Species	Transgene	Comments [ref]
Tet-Off (VC1)	Dox (300 *μ*g/mL and 2 mg/mL)	AAV	Rat	GFP	Some leakiness. No further transgene repression by increasing dox dosage [4].

Tet-On (VC2)	Dox (6 g/kg)	AAV	Rat	EGFP	No repression or basal expression data [5].

Tet-On (VC1 & 2)	Dox (2 mg/mL)	Ad	Rat	LacZ	Negligible expression following dox removal [6].

Tet-Off (VC1)	Dox	AAV	Rat	GDNF	Undetectable transgene levels at serum dox levels below those required for antimicrobial activity [7].

Tet-Off (VC2)	Dox (300 *μ*g/mL)	AAV	Rat	EGFP	Low basal expression with addition of insulator sequences [9].

Tet-On (VC2)	Dox (600 *μ*g/mL)	AAV	Rat	GDNF	Some basal expression [11].

Tet-Off (VC3)	Dox (2 mg/mL)	Ad	Rat	Caspase-9 & EGFP	Variable repression achieved; transgene dependent [13].

Tet-On (VC1)	Dox (2 mg/mL)	LV	Rat	Luciferase	Low basal expression [19].

Rapamycin	Rapamycin (10 mg/kg)	AAV	Rat	hAADC	Robust phenotypic response in 6-OHDA model. Low basal expression without phenotypic effect [25].

Rapamycin	AP21967 (1 mL/kg)	LV	Mouse	GFP & GDNF	No basal expression [28].

RU486	RU486 (25 mg/kg)	HSV	Rat	LacZ	Very low basal expression [31].

HRE	30 min hypoxia in a 3% O_2_ hypoxic chamber	Ad	Mouse	Luciferase	No transgene expression under normoxic conditions [36].

HRE	60 min MCAO	Ad	Mouse	BDNF & EGFP	Neuroprotection & phenotypic changes seen. No comparison with unregulated transgene expression [37].

HRE	45 min MCAO	AAV	Mouse	VEGF or LacZ	Angiogenesis seen. No transgene expression under nonischemic conditions [39].

HRE	Spinal cord contusion injury	Naked plasmid injection	Rat	VEGF	Locomotor recovery compared to uninjected controls [42].

HRE	Spinal cord contusion injury	Naked plasmid injection	Rat	Luciferase	Higher transgene expression in injured compared to normal spinal cord [44].

HRE	Spinal cord contusion injury	*Ex vivo* stem cell therapy	Rat	Luciferase	Low transgene expression in normal spinal cord [45].

*VC, Vector Configuration (see Figure 2); Dox, doxycycline; GFP, Green Fluorescent Protein; EGFP, Enhanced Green Fluorescent Protein.
